# The Importance of Sex Differences on Outcome after Major Trauma: Clinical Outcome in Women Versus Men

**DOI:** 10.3390/jcm8081263

**Published:** 2019-08-20

**Authors:** Julian Joestl, Nikolaus W. Lang, Anne Kleiner, Patrick Platzer, Silke Aldrian

**Affiliations:** 1 Department of Orthopedics & Trauma Surgery, Medical University of Vienna, Waehringer Guertel 18-20, A-1090 Vienna, Austria; 2 Department of Orthopedics and Traumatology, Landesklinikum Baden-Moedling-Hainburg, A-2340 Moedling, Austria

**Keywords:** Polytrauma, Multiple trauma, Sex related differences, Outcome, ARDS, MOF

## Abstract

Purpose: The purpose of this study was to evaluate epidemiological and clinically relevant sex-related differences in polytraumatized patients at a Level 1 Trauma Center. Methods: 646 adult patients (210 females and 436 males) who were classified as polytraumatized (at the point of admission) and treated at our Level I Trauma Center were reviewed and included in this study. Demographic data as well as mechanism of injury, injury severity, injury pattern, frequency of preclinical intubation, hemodynamic variables on admission, time of mechanical ventilation and of intensive care unit (ICU) treatment, as well as the incidence of acute respiratory distress syndrome (ARDS), multi organ failure (MOF), and mortality were extracted and analyzed. Results: A total of 210 female and 436 male patients formed the basis of this report. Females showed a higher mean age (44.6 vs. 38.3 years; *p* < 0.0001) than their male counterparts. Women were more likely to be injured as passengers or by suicidal falls whereas men were more likely to suffer trauma as motorcyclists. Following ICU treatment, female patients resided significantly longer at the casualty ward than men (27.1 days vs. 20.4 days, *p* = 0.013) although there was no significant difference regarding injury severity, hemodynamic variables on admission, and incidence of MOF, ARDS, and mortality. Conclusion: The positive correlation of higher age and longer in-hospital stay in female trauma victims seems to show women at risk for a prolonged in-hospital rehabilitation time. A better understanding of the impact of major trauma in women (but also men) will be an important component of efforts to improve trauma care and long-term outcome.

## 1. Introduction

Although the clinical management algorithm for polytraumatized patients has recently improved considerably, injury remains the leading cause of death and severe disability in the adult population [1,2]. Standardized pre-hospital treatment protocols at the scene, resuscitation guidelines in the emergency room (ER), as well as intensive care and rehabilitation management provide equal principles for all patients, irrespective of the sex. Due to a better scientific knowledge of the complex pathophysiological and immunological response following severe trauma, tremendous changes in the overall care of multiple-injury patients have evolved [3,4]. In this context, sex related differences are increasingly being recognized and might eventually be incorporated into future management guidelines. There is emerging evidence suggesting that males and females respond in different ways to severe injury, not only due to the anatomic differences. Previous studies investigating sex-related differences in trauma patients have focused on sex itself as a risk factor for poor in-hospital outcomes such as sepsis and multiple organ failure (MOF).

The aim of this study was to evaluate epidemiological and possible clinically relevant sex differences in multiple-trauma patients at a Level I Trauma Center.

## 2. Materials and Methods

We retrospectively searched our department’s trauma database for all adult (18 years or older) polytraumatized patients admitted to the hospital for at least one day, as well as all patients declared dead in the Department of Trauma Surgery. Polytraumatized was defined as injuries of two or more body cavities, or injury of one body cavity and two long bone fractures with an Injury Severity Score (ISS) of ≥16. Data sets of demographic data, injury severity, injury pattern, types of accidents, frequency of preclinical intubation, hemodynamic variables on admission, duration of mechanical ventilation and intensive care unit (ICU) treatment, and incidence of acute respiratory distress (ARDS), MOF, and mortality were extracted and reviewed. Systemic hemodynamic variables on admission were defined as unstable if the systolic blood pressure decreased ≤90 mmHg. Injury severity was classified according to the Abbreviated Injury Scale (AIS) and Injury Severity Score (ISS) [5,6,7,8,9,10,11,12,13,14,15,16,17,18]. MOF was scored with ≥2 points for two or more failing organ systems (pulmonary, cardiovascular, hepatic, renal, central nervous, hematological, and gastrointestinal systems) over a period of three days or longer, according to Goris et al. [19]. ARDS was defined as a PaO_2_/FiO_2_ ratio <200 for at least five consecutive days and bilateral diffuse infiltrates seen on the chest X-ray in the absence of pneumonia and cardiogenic pulmonary edema [20]. Patients with isolated, severe, and potentially life-threatening injuries, as well as patients younger than 18 years were excluded from this study. The data set was handled anonymously. The study was approved by the Institutional review board and was performed in accordance to the Declaration of Helsinki.

The statistical analysis comparisons of mean values between groups were performed retrospectively with Wilcoxon-two-sample-tests. Comparisons of dichotomous variables were tested with the Fisher’s Exact Test. To check for associations with hemodynamic, reanimation rate, ARDS, MOF, and mortality, we constructed logistic regression models for sex as an independent variable and age as the covariate. An analysis of covariance was done to compare mean values with sex as an independent variable and age as the covariate. Stepwise logistic regression was used to examine the influence of sex on ventilation time and mortality rate with age, injury severity, and hemodynamic parameters as independent variables to model the probability of death. To calculate the duration of ventilation, a stepwise Cox-model with the event alive-release was performed. If a patient died, the ventilation time was censored with the maximum (63 days). All *p* values < 0.05 were considered as statistically significant. Due to the retrospective design, all analyses had explorative character and no correction for multiplicity was done. Metric variables (e.g., patient’s age) are reported as mean ± SEM (standard error of mean), and categorical data as numbers and percentages. SAS statistical software system (Version 8.2, SAS Institute Inc., Cary, NC, USA) was used to carry out the statistical analyses.

## 3. Results

**Figure 1:**
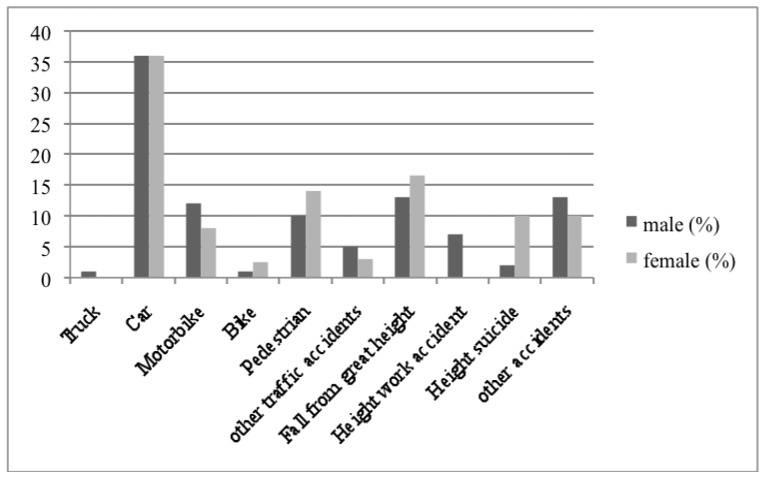
Trauma events.

Six hundred forty-six polytraumatized adult patients who had been admitted to our Level I Trauma Center between 2000 and 2017 formed the basis of our report. The majority of patients were male (*n* = 436 (67.5%)) with a mean age of 38.3 ± 0.7 years (range: 18.9 to 92.3) vs. 44.6 ± 1.3 years (range: 18.0 to 90.7) in females (*p* < 0.0001). The sex-related differences in mechanism of injury are listed in Figure 1. Suicide related falls from great heights and accidents as pedestrians were seen more frequently in females, whereas the majority of injured motorcyclists were male; these differences were not significant.

### 3.1. Injury Severity and Injury Pattern

With regard to injury severity, the mean ISS of the male study population was 34.6 ± 0.6 (range: 19 to 75) vs. 33.8 ± 0.9 (range: 18 to 75) in the female group (*p* = 0.979). A significant increase in trauma severity was seen with increasing age (*p* = 0.001). Table 1 shows sex-related injury patterns according to the abbreviated injury score (AIS) by body region. We found a greater abdominal injury severity in men and more severe trauma of the spine, extremities, and the pelvis in women; these differences were not significant. The most severe injuries occurred in the chest region of both groups.

### 3.2. Preclinical Features

With regard to preclinical features (Table 2), preclinical intubation and ventilation rates were 70.6% in female patients and 68.9% in male patients, with no significant differences. In total, 29.2% of female trauma victims and 25.5% of male patients presented with unstable hemodynamic conditions at Emergency Department arrival; this difference was not significant and not influenced by the patients’ age (*p* = 0.562). Likewise, resuscitation rate on admission was similar in both groups and did not show significant sex or age-related differences (*p* = 0.354).

### 3.3. Intensive Care Therapy and Course

Table 3 compares the mean duration of ventilation, the length of stay in the intensive care unit and the casualty ward, along with the total hospitalization between males and females. The results showed no statistically significant difference between the groups. However, following intensive care management, female patients showed a statistically significant longer residence time at the ward (*p* < 0.001); this difference was not influenced by the patient’s age (*p* = 0.612).

### 3.4. Post-Traumatic Complications and Mortality

Significant sex-related differences concerning the incidence of ARDS (females: 11.4%; males: 13.8%; *p* = 0.348) and MOF (females: 9.0%; males: 7.6%; *p* = 0.919) were not evident, but the incidence of MOF significantly increased with the patient’s age (*p* = 0.001). The difference in hospital mortality between 27.0% of female and 32.5% of male patients did not show significance (*p* = 0.657). However, a significant increase in hospital mortality was seen with increasing age (*p* = 0.003).

## 4. Discussion

Similar to the recent exploration of sex-related differences in many specialties of medicine, the influence of sex differences has been of great interest in the treatment of polytraumatized patients. The proportion of female trauma victims is on the rise. A retrospective analysis of polytrauma management (1975–2004) revealed a significant decrease of the male to female ratio from 2:1 to 1:1 within the last decade (1995–2004) [21]. The results of the present study of a cohort of patients ≥18 years of age indicated a significantly higher mean age of the female trauma victims compared to their male counterparts, however, more male than female patients were affected (436 to 210 patients). This is in contrast with the results of a clinical re-examination study of polytraumatized patients published by Probst et al. [22] demonstrating no sex-related differences in age. This study, however, included a cohort of trauma victims from 3 to 60 years-of-age at the time of injury. Although trauma mechanisms in the present study were not significantly different between the sexes overall, a distinct pattern became apparent in female patients. Consistent with the findings of other studies, men were more likely to be injured as motorcyclists vs. females, who were more likely to be injured as pedestrians or by suicidal jumps [4,23]. Aufmkolk et al. [24] studied the incidence of accidental versus intentional falls from great heights in multiple trauma patients and reported a higher number of female patients to be injured by suicide-related falls from great heights. In addition, the injury pattern after intentional falls more often involved fractures of the lumbar spine, pelvis, and lower extremities [24]. A similar sex related specific pattern connecting trauma mechanism and injury pattern was found in the present study, as the elevated rate of severe spine, pelvis, and extremity injuries in the female group was associated with a higher number of suicide-related falls. As a preclinical feature, injury severity was similar in both sexes. The female trauma patients were more often hemodynamically unstable on admission than were male patients, however, this might be due to the fact that male patients more often died directly after suicidal falls, as they tend to choose greater heights to successfully die. This disparity might be explained by a weakness of the ISS, which is solely based on anatomic criteria and does not incorporate vital signs and/or clinical parameters. In addition, the higher mean age of our female study population might have contributed to the unstable clinical condition, as advanced age has been proven to be a significant risk factor in the acute setting [25,26]. Clinical studies examining the effect of sex-related differences on morbidity and mortality following trauma yielded conflicting results in the past. In the present study, polytrauma was not significantly related to the incidence of multi organ failure, acute respiratory distress syndrome, or survival in a sex-related specific way. This is in accordance with several other studies that did not find any divergence in male and female trauma outcomes as well [27,28,29,30]. Bowls et al. [27] analyzed the long-term-outcome in 15,170 trauma victims (retrospective review) and identified age, injury mechanism, and injury severity, but not sex relation as factors influencing survival. Gannon and colleagues prospectively examined the effect of female sex on outcome in 22,332 trauma patients. After adjusting the variables known to affect outcome after trauma (e.g., ISS and age) female sex did not predict in-hospital mortality [30]. Offner et al. [31] studied moderately to severely injured patients and found no difference in mortality rates relating to sex, but noted that male trauma patients had an increased risk of major infections. Furthermore, the results of the current study are not consistent with studies that suggest that sex-related differences in outcome following severe trauma do exist. Frink et al. [13] in a prospective study, investigated the effect of sex and age on organ dysfunction and the clinical course in 143 patients with multiple injuries. Sex differences were confirmed in incidence and outcome of MOF, with a benefit observed in females. Premenopausal women with an ISS greater than 25 suffered significantly less MOF compared with age-matched males. Wohltmann et al. [10] reviewed >20,000 consecutive admissions to Level I Trauma Centers and found that males younger than 50 years old had increased mortality rates compared with females. In this study, young injured men showed a 27.0% greater chance of dying than injured women. Taken together, significant controversies about sex-related differences in long-term outcome after polytrauma still exist.

One of our major findings was the significantly longer stay of female polytraumatized patients at the casualty ward. Holbrook et al. [3] examined sex-related differences in short- and long-term functional outcomes of 1048 major trauma patients and demonstrated a strong and independent role of sex in predicting functional outcome and quality of life after severe trauma. Functional outcome was significantly worse at each follow-up time in females. Gannon et al. furthermore demonstrated in a retrospective study that traumatized patients between 46 and 64 years-of-age stayed significantly longer in the hospital than the younger group [30]. The positive correlation of age and hospital length of stay in female trauma victims of our study may put women at risk for a less efficient prolonged in-hospital rehabilitation time.

## 5. Limitations

There are several limitations to this study. We retrospectively evaluated a small sample size, analyzing an inhomogeneous patient population in a single-center designed study. Further prospective studies with larger numbers and longer follow-up are required to investigate the long-term outcome.

## 6. Conclusions

The correlation of higher age and longer stay of female polytraumatized patients at the casualty ward seems to show women at risk for a prolonged in-hospital rehabilitation time. This fact requires more attention in the future. Potential complex interactions of additional factors (e.g., sex hormones) preclude a precise statement about the impact of sex related differences on management of polytrauma and long-term outcome. Future studies should undoubtedly account for the hormonal status of female patients to accurately assess the role of sex-related differences in trauma patients.

## Figures and Tables

**Table 1:** Injury Severity Scores.

Region	Sex	Mean AIS	SD	SEM	*p-*Value
Head	male	2.41	1.9	0.091	0.825
	female	2.38	1.9	0.130	
Neck	male	0.08	0.5	0.022	0.988
	female	0.08	0.4	0.028	
Chest	male	2.79	1.6	0.079	0.490
	female	2.70	1.7	0.116	
Abdomen	male	1.63	2.0	0.095	0.240
	female	1.43	1.9	0.133	
Spine	male	0.69	1.3	0.064	0.212
	female	0.83	1.6	0.102	
Extremities, Pelvis	male	2.19	1.4	0.065	0.236
	female	2.32	1.4	0.094	

**Table 2:** Preclinical Data.

Preclinical	Female		Male		*p-*Value
	*number*	%	*number*	%	
Intubation rate	149	70.6%	303	68.9%	0.321
Hemodynamically unstable	61	29.2%	109	25.4%	0.325
Resuscitation	15	7.2%	29	6.7%	0.634

**Table 3:** Intensive Care Data.

Duration of	Sex	Days	SD	SEM	*p-*Value
Ventilation	Male	15.0	28.0	1.368	0.828
	female	16.4	39.0	2.767	
Intensive care unit	Male	18.4	24.1	1.167	0.359
	female	17.3	17.1	1.200	
Casualty ward	Male	20.4	27.2	1.328	0.013
	female	27.1	32.4	2.292	
Total hospitalization	Male	39.0	39.8	1.933	0.178
	female	44.3	39.6	2.800	
